# Advances in Modeling Polyglutamine Diseases Using Genome Editing Tools

**DOI:** 10.3390/cells11030517

**Published:** 2022-02-02

**Authors:** Marianna Karwacka, Marta Olejniczak

**Affiliations:** Department of Genome Engineering, Institute of Bioorganic Chemistry, Polish Academy of Sciences, 61-704 Poznan, Poland; marianna.karwacka@gmail.com

**Keywords:** CRISPR-Cas9, genome editing, disease models, polyglutamine disease, polyQ, CAG repeats, iPSCs, Huntington’s disease

## Abstract

Polyglutamine (polyQ) diseases, including Huntington’s disease, are a group of late-onset progressive neurological disorders caused by CAG repeat expansions. Although recently, many studies have investigated the pathological features and development of polyQ diseases, many questions remain unanswered. The advancement of new gene-editing technologies, especially the CRISPR-Cas9 technique, has undeniable value for the generation of relevant polyQ models, which substantially support the research process. Here, we review how these tools have been used to correct disease-causing mutations or create isogenic cell lines with different numbers of CAG repeats. We characterize various cellular models such as HEK 293 cells, patient-derived fibroblasts, human embryonic stem cells (hESCs), induced pluripotent stem cells (iPSCs) and animal models generated with the use of genome-editing technology.

## 1. Introduction

Polyglutamine (polyQ) diseases belong to a group of progressive neurodegenerative disorders. The defining characteristic of polyQ diseases is the presence of an expanded CAG repeat tract in the coding sequences of various genes (Table 1). The polyQ expansions are fundamental to nine genetic neurological diseases, namely Huntington’s disease (HD), spinocerebellar ataxias (SCAs) (SCA1, SCA2, SCA3, SCA6, SCA7 and SCA17), dentatorubral-pallidoluysian atrophy (DRPLA) and spinobulbar muscular atrophy (SBMA) [1,2,3].

PolyQ diseases share major features, such as the abnormal expansion of CAG repeats within exonic sequences and aggregation of structurally aberrant proteins. It remains under discussion whether the abnormal protein structure leads to neurodegeneration by the toxic gain of function, by the loss of function or by a combination of these two mechanisms [4,5]. Recently numerous studies have proven that the gain of function contributes to the self-assembly of the protein into toxic aggregates and the generation of neuronal intranuclear inclusions (NIIs), leading to neuronal death [6,7,8,9,10,11,12,13]. However, the neurodevelopmental significance of mutated genes supports the loss-of-function theory as the altered protein products may contribute to improper neural functioning [5].

Clinically, all polyQ diseases exhibit threshold phenomena. Progressive neurological features are observed once repeat numbers exceed disease-specific limits. Additionally, trinucleotide tracts are unstable and increase their length when transmitted to the next generations. A higher number of repeats causes an earlier and more severe disease phenotype [14,15]. CAG repeats increase their copies gradually and not only in germline cells. Somatic expansions, which arise from a defective mismatch repair, occur in a tissue-specific manner in various organs and regions of the brain [16,17,18].

Despite having similar features, polyQ diseases differ regarding their altered genes and protein products, the number of pathogenic CAG repeats, the preferentially degenerated neuronal subpopulations and the affected brain regions [1,2,6,19] (summarized in Table 1).

Modern, nuclease-based gene-editing methods such as meganucleases, zinc finger nucleases (ZFNs), transcription activator-like effector nucleases (TALENs) and clustered, regularly interspaced, short palindromic repeats (CRISPR)-Cas9 technology have significantly contributed to advances in disease modeling and augmented a variety of scientific research. Since their invention, genome-editing tools have become increasingly popular due to their ability to directly change DNA sequences and alter gene expression efficiently [40,41].

Here, we describe polyQ disease models’ application to determine which genome-editing methods were applied, considering the existing models based on non-genome-editing techniques. Application of modern genome-editing methods and development of new, more relevant models valuably contributes to improving our knowledge of polyQ diseases and lays a promising foundation for future therapeutic strategies.

## 2. Genome-Editing Tools

The majority of editing approaches involve the generation of a double-strand break (DSB) in DNA and activation of repair systems: either non-homologous end joining (NHEJ) or homology-directed repair (HDR) [40,42]. NHEJ is an efficient but imprecise process in which the chromosome ends are joined back together while introducing small insertions and deletions (INDELs), resulting in the frameshift mutation and knockout of the protein-coding gene [43]. HDR uses a homologous sequence as a template to precisely repair the cleavage site, and in the presence of an extrachromosomal donor, can be applied to introduce specific changes in the DNA sequence [44,45,46,47]. This method has been used successfully for the generation or correction of disease-specific mutations.

### 2.1. Genome Editing before the CRISPR Era

One of the first described systems of genome editing was restriction enzymes called meganucleases. They were first discovered in yeast and subsequently used to generate insertions, deletions and frameshift mutations [48]. Meganucleases have not been used to model any of the polyQ diseases. The application of meganucleases is hampered by the difficulty in matching the recognition site with the targeted loci. In most cases, the recognition sequence, which ranges from 14 to 40 base pairs, must be designed and inserted in the target site [49,50].

ZFNs, described in 1996 by Kim et al., are composed of a zinc finger DNA-binding domain and the catalytic domain of a FokI endonuclease [51]. Specific DNA recognition of 18 base pairs is provided by three to four zinc finger protein domains (ZFPs), each binding to three nucleotides [49]. The limitations of this method are the reduced efficiency at the targeted site resulting from the chromatin organization or epigenetic changes, as well as potential immunogenicity and a high off-target probability [47,49]. The advancement in this technology was the introduction of different effector domains, allowing for gene activation (VP64), silencing (KRAB) or methylation (DNMT1) [52]. No polyQ models have been created using ZFNs, but this technology was used to reduce huntingtin expression in the R6/2 mouse brain [53] and to study CAG repeats’ instability in cell lines [54,55].

TALENs contain a programmable DNA-binding domain with 34 amino acid repeated units fused with a DNA-cleavage domain of the FokI enzyme [56,57]. The highly variable amino acids on the 12th and 13th positions, called repeat-variable diresidues (RVDs), are responsible for nucleotide recognition [46,52,58]. The lengths of target sequences for TALENs extend from 50 to 60 bp (including a 14–18 bp spacer, where FokI acts as a dimer and cleaves opposite binding sites for paired TALENs) [59]. On the contrary, ZFPs bind to three nucleotides, and RVDs interact with individual DNA base pairs, which considerably increases the number of targetable sequences [49,57]. However, due to DNA methylation and acetylation of histones, TALENs interact poorly with inactive chromatin [60]. Additionally, this system requires thymidine at the 5′ end of the target site, which limits the choice of target sequence [61]. TALENs were used to model HD in induced pluripotent stem cells (iPSCs) and human embryonic stem cells (hESCs) [62,63]. Additionally, technology based on TALENs was used for allele-specific reduction of mutant huntingtin in human fibroblasts [64] and to induce contractions of the CAG tract in yeast [65].

### 2.2. CRISPR-Cas9 System

The CRISPR-Cas9 technology developed in 2012 significantly increased the availability and use of genome-editing methods in science. Their simplicity of targeting, efficiency and ability to simultaneously target multiple sites made CRISPR-Cas9 tools the most powerful and flexible approaches to precise genome editing and targeted gene regulation [66].

The CRISPR-Cas9 system is composed of guide RNA (gRNA) and a Cas9 nuclease, which induces DSBs in the genome [66,67,68]. Contrary to previously described methods, the targeting specificity of the CRISPR-Cas9 complex is determined by RNA-DNA interactions rather than by protein recognition [67]. The target site, which is approximately 20 bp, is determined by the presence of a protospacer-adjacent motif (PAM) at the 3′-end and identified by complementary gRNA [69]. The natural diversity of PAM sequences recognized by the Cas proteins from different bacterial species considerably enhances the targeting scope of CRISPR-mediated genome editing [61,70].

The biggest concern regarding CRISPR-Cas9 technology is the off-target effect, as Cas9 nucleases tolerate mismatches depending on their distribution and number. There have been two main approaches to minimizing off-target events. The first approach is to reduce the exposure time of DNA to Cas9 by reducing the nuclease concentration, delivering the protein in the ribonucleoprotein (RNP) complex or using an inducible Cas9. The other involves engineered Cas variants, such as the recently proposed Sniper-Cas9, with increased sensitivity and target specificity [71,72].

The application of the CRISPR system has been further extended by the use of other effector nucleases such as the Cas12a protein or by using modified nucleases such as Cas9 nickase (Cas9n), which only cleaves one strand at the target site, or catalytically-dead Cas9 (dCas9), which loses its nuclease activity but maintains the ability to bind to the sequence targeted by gRNA [66,73,74].

The CRISPR-Cas9 method is widely used both in modeling polyQ diseases and in research related to the precise removal of the CAG expansions. To date, CRISPR-Cas9 technology has been used to modify the CAG tract in HD patient-derived fibroblasts [69,75,76], human HD neural progenitor cells (NPCs) [76], HD patient-derived iPSCs [76], mesenchymal stem cells (MSCs) extracted from the bone-marrow of YAC128 mice [77] and a BacHD mouse model [69].

## 3. Approaches to Modeling PolyQ Diseases

PolyQ diseases affect neural tissue, which is extremely difficult to obtain from patients. Therefore, cellular and organism models are necessary for research in this subject area. PolyQ models are generated according to two approaches: the first is the introduction of polyQ expansions, and the other is the correction of existing expansions to generate isogenic controls. 

Rangel-Barajas et al. proposed dividing HD mouse models into three categories [78], and this classification can be extrapolated for all models with pathological polyQ expansions. The first category is truncated models with polyQ-coding fragments, such as the R6/2 line bearing solely exon 1 of the mutated *HTT* gene, which successfully generates a progressive neurological phenotype and shows neurodegeneration [79,80]. As this example demonstrates, the most frequently used polyQ models contain only a gene fragment with a CAG tract. The second group is represented by models with the full human mutant gene. For instance, full-length HD rodent models were created using yeast artificial chromosome (YAC) [81,82] and bacterial artificial chromosome (BAC) technology [83,84]. Knock-in models with mutant genes inserted in the proper genetic context belong to the last category and are believed to best reflect the human genetic condition [78,85,86]. Accordingly, models with polyQ expansions are a good means of studying pathological phenotypes, such as protein aggregation, changes in cell morphology and neurodegeneration, as well as for testing possible therapeutic approaches. Additionally, the models explore genetic pathways and mechanisms underlying disease development such as expansions, aberrant splicing, repeat-associated non-AUG (RAN) translation and RNA frameshifting. By using methods such as lentiviral transduction, microinjection with mRNA and transposase-mediated recombination, transgenic models were generated in yeast [87,88], human embryonic kidney (HEK) 293T cells [89,90,91], *C. elegans* [92,93,94,95], *D. melanogaster* [96,97,98], zebrafish [99,100,101,102], the mouse [27,103,104,105,106,107,108,109,110,111,112,113], rat [83,114], sheep [115], pig [116,117] and monkey [118,119,120,121]. Additionally, patient-derived cells were reprogrammed into iPSCs and used to model polyQ diseases [122,123,124,125,126,127,128]. Nowadays, with the development of new genome-editing tools, CRISPR-Cas9 technology is most often used for modeling.

The second trend in modeling polyQ diseases involves the correction of expanded CAG repeat tracts. Models with contracted polyQ expansions are used as isogenic controls against the cell from which they were derived. It is vital to exclude interference caused by different genetics and epigenetics [129] as subtle differences in DNA may influence somatic instability or disease onset [130]. Isogenic models of polyQ disease were generated by targeted HR [131] and genome-editing tools, mainly CRISPR-Cas9 technology [130,132,133,134,135,136,137,138,139].

## 4. Cellular Models of polyQ Diseases

Cellular models have contributed to the discovery and validation of many pathological changes related to polyQ diseases. They are an indispensable element in research as they allow for more rapid, economical and highly-controlled experiments.

### 4.1. Fibroblasts

PolyQ cell models with clinically relevant phenotypes can be obtained by culturing fibroblasts extracted from patients by a skin biopsy [140,141,142,143]. Fibroblast-based models are often used in polyQ research but are difficult to transfect with plasmids and sometimes show low expression of polyQ genes. Primary fibroblasts are prone to telomere-controlled senescence, which restricts multiple passages and their long-term use [144]. Hung et al. generated hTERT-immortalized HD fibroblasts, which gain proliferative capacity and remain stable during long-term experiments [144]. Despite the utility of immortalized cell lines, their genome might undergo some changes in its structure and copy number [145].

Goetz et al. point out the importance of carefully selecting age- and sex-matched controls, choosing the same regions of the body for biopsies and treating all obtained cells identically [141]. Yet, controls from different individuals vary in terms of their genetic background, which may affect the observed results [130]. This problem can be resolved by CRISPR-Cas9 genome editing, which allows for the generation of isogenic controls.

The advantage of fibroblasts is that they can be reprogrammed into iPSCs, which can be further differentiated into neurons [144].

Malankhanova et al. generated human embryonic fibroblasts with heterozygous insertion of 69 CAG repeats in the first exon of the *HTT* gene using CRISPR-Cas9-mediated HR [146]. The engineered model was tested for the presence of CAG expansions through PCR screening, western blot analysis and capillary electrophoresis. The off-target sites selected by in silico analysis were examined by Sanger sequencing and revealed no undesirable genomic modifications. Both the unmodified fibroblasts and genome-edited mutants were then reprogrammed to iPSCs using episomal vectors bearing pluripotency factors OCT4, KLF4, L-MYC, SOX2 and LIN28. Characterization of iPSCs involved karyotyping, analysis of the NANOG, OCT3/4 and SOX2 gene expression, immunofluorescence staining for pluripotency markers and an embryoid bodies formation assay. The tests proved the pluripotent nature of the cells and their correct chromosomal composition. The mutant iPSCs, wild-type isogenic control and a non-isogenic positive control were directly differentiated into medium spiny neurons (MSNs), confirmed by immunofluorescence staining of MSN markers. Both the positive control 47-CAG iPSCs line and the edited 69-CAG iPSCs line showed impaired neural rosette formation in comparison to the healthy control. Caspase 3 immunostaining revealed increased neuronal death after the growth factor withdrawal in positive control and mutated MSNs (Table 2).

However, in agreement with the studies of An et al. (2012), the mutated and patient-derived MSNs did not develop characteristic huntingtin- or polyQ-positive aggregates [131]. Electron microscopy of mutant MSNs with 69 CAG repeats showed various ultrastructural defects in comparison with the unmodified MSN isogenic control. Neurons with HD displayed dense cytoplasm, abnormal organelle organization and structure and atypical dendrites, spines and synapses.

### 4.2. Embryonic Stem Cells

**Figure 1:**
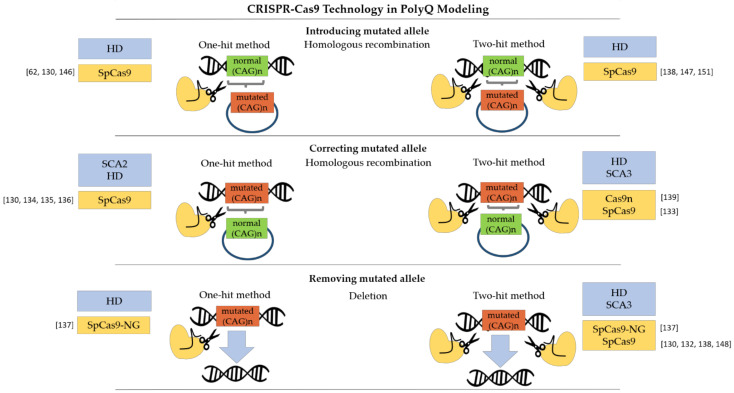
Overview of approaches to creating polyQ disease models with CRISPR-Cas9 genome-editing tool. CRISPR-Cas9 technology is used to introduce or repair a mutant allele by inducing homologous recombination (HR), as well as to excise a mutant DNA sequence. In either case, single-molecule (one-hit method) [62,130,134,135,136,137,146] or two-molecule (two-hit method) [130,132,133,137,138,139,147,148,151] gRNA may be used. The use of modified *Streptococcus pyogenes* Cas9 (SpCas9) nucleases further enhances the modeling possibilities of polyQ diseases.

Patient-derived ESCs contain disease-related genetic patterns and can be differentiated into any cell in the human body. Additionally, they possess the natural ability to divide endlessly, so there is no need to induce cancerous modifications [145]. These cells can be used to study the molecular mechanism of polyQ diseases and generate disease-specific neuronal models. An overview of the methods for polyQ models’ generation with the use of CRISPR-Cas9 technology is presented in Figure 1. Ruzo et al. (2018) and Ooi et al. (2019) used targeted endonucleases together with a Piggy-Bac selection system to create a panel of isogenic ESCs with different lengths of CAG expansions in exon 1 of *HTT* [63,138]. The first group used CRISPR-Cas9-mediated HR, whereas the second group used TALEN-mediated HR. Additionally, Ruzo et al. prepared heterozygous and homozygous *HTT* knockout mutants with CRISPR-Cas9 technology. Both collections of HD models were confirmed to retain their pluripotency and a normal karyotype. Genome-wide copy number variation (CNV) analysis did not detect any CNVs. To confirm the correct knock-in of the expanded CAG, PCR [63,138] or *HTT* allele sequencing [138] was performed. Ooi et al. did not analyze the potential off-targets, unlike Ruzo et al., who performed whole-genome sequencing and found no mutations in any of the top-five predicted off-target loci.

Ruzo et al. differentiated the collection of ESC models into neurons and revealed that both expansion of the polyQ fragment and lack of the *HTT* gene resulted in a reduced fraction of larger neural rosettes, decreased self-organization of neural rosettes and dysregulation of mitotic spindle orientation. Thus, they concluded that in a developmental context, HD is caused by a loss of function mechanism leading to chromosomal instability that impairs neurogenesis [138]. Ooi et al. showed by western blot analysis that *HTT* expression decreases with increasing CAG repeat length. Their experiment also confirmed the differentiation potential of the created ESCs by developing NPCs, neurons, hepatocytes and myotubes. NPCs presented HD-related phenotypes such as deficits in mitochondrial function, elevated reactive oxygen species (ROS), increased susceptibility to DSBs and alterations in cell proliferation. Genome-wide sequencing and proteomics assays revealed transcriptional differences between both CAG lengths and cell types [63].

### 4.3. Induced Pluripotent Stem Cells

Cellular models that connect the advantages of ESCs and patient-derived fibroblasts are iPSCs. iPSCs contain patient-specific genetic information, divide unlimitedly and can be differentiated into any disease-relevant cell populations, including neurons [152,153]. In their undifferentiated form, iPSCs can be a good model for studying molecular changes typical of the early phenotypes of polyQ diseases, such as gene expression, cellular signaling and formation of aggregates [153]. They provide an accessible platform for studying disease mechanisms and allow for drug screening. Park et al. were the first to generate the iPSCs model of polyQ disease by reprograming fibroblasts from HD patients [122]. Until now, all polyQ diseases except for SCA17 have been modeled in iPSC lines [6,19,154]. However, no phenotypic studies have been carried out yet for DRPLA, SCA1 and SCA7 iPSCs models [6].

Despite the undeniable advantages of iPSCs and iPSC-derived cells, the reprogramming process induces genetic instability and changes epigenetic signatures back to the immature fetal stage [153]. Karyotypes of generated iPSCs should always be analyzed, as HD iPSCs models of HD have revealed certain chromosomal aberrations [155]. Additionally, reprogramming and differentiation of iPSCs is a cost-inducing and time-consuming process [156]. The use of genome-editing methods favors further adaptation of polyQ models to the needs of the experiment. An et al. were first to correct iPSCs derived from HD patient fibroblasts by using HR, creating the basis and guideline for further research in this field [131]. iPSC lines were subsequently corrected using CRISPR-Cas9 technology to generate isogenic HD [130,139], SCA2 [134,135,136] and SCA3 models [132,133].

In 2016, Marthaler et al. used CRISPR-Cas9 technology to replace the expanded CAG region in the *ATXN2* with a wild-type 22-CAG repeat fragment in three iPSC lines (H271, H266, H196) from previously reprogrammed SCA2 patient skin fibroblasts [134,135,136]. All models were characterized by PCR genotyping and Sanger sequencing, tested positive for expression of pluripotency markers by RT-qPCR and immunocytochemistry and confirmed the correct karyotype by G-banding. All of the isogenic SCA2-iPSCs lines remained pluripotent and maintained the potential to differentiate into cell types of the three germ layers. The efficiency of this CRISPR-Cas9-mediated modification remains unknown and no further experiments were performed on the generated models.

In 2017, Xu et al. created an isogenic model with 18 CAG repeats from HD iPSCs with 180 CAG repeats using Cas9n, a pair of gRNAs and Piggy-Bac system for selection [139]. Later, in 2020, Dabrowska et al. used CRISPR-Cas9-mediated HR to replace 119 CAG repeats with 19 CAG repeats in exon 1 of the *HTT* gene in the iPSC model of juvenile HD [130]. Unlike the protocol used by Xu et. al., the approach taken by Dabrowska et al. did not require additional selection. Yet, they both achieved a similar efficiency of 5/6%. The generated isogenic control iPSCs were characterized by Sanger sequencing. The models were confirmed to be free of possible off-targets, to have a normal karyotype and to remain pluripotent. In addition, a double knockout iPSC model of HD was generated by Dabrowska et al. using a pair of gRNAs and Cas9n, and was confirmed by a western blot [130]. Xu et al. showed that corrected iPSCs can be differentiated into NPCs and further into excitable, synaptically active GABAergic neurons. Isogenic iPSC-derived neural cells had an ameliorated disease phenotype in terms of neural rosette formation, susceptibility to growth factor withdrawal and mitochondrial respiration [139]. Global differential gene expression analysis in iPSCs and NPCs showed no significant differences between HD and corrected lines [139]. This underlines the fact that the introduction of isogenic controls into the panel of cells used in comparative studies is necessary as most differences may be related to the genetic background rather than HD-specific aspects.

A perfect example of an isogenic model’s application is a study conducted by Pourshafie et al., in which they used CRISPR-Cas9-mediated knockout of the AR gene in SBMA patient-derived iPSCs and healthy control models [157]. The cells were further differentiated into motor neurons to examine epigenetic dysregulation of metabolic genes and its link to mitochondrial impairment during SBMA pathogenesis. The use of isogenic knockout controls allowed the researchers to distinguish the effects of the AR toxic gain-of-function phenotype in the cells from loss-of-function. Interestingly, contrary to the SBMA models, AR knockout motor neurons retained their ability to regulate bioenergetic homeostasis despite their compromised mitochondrial activity.

Two SCA3 iPSC models with corrected CAG repeats in exon 10 of the *ATXN3* gene have been created so far [132,133]. Ouyang et al. (2018) excised the CAG repeat tract by using CRISPR-Cas9 technology with paired gRNAs to promote the production of a truncated *ATXN3* protein without the toxic polyQ domain [132]. Based on PCR screening and Sanger sequencing, they chose clones with an unaffected normal allele and mutated allele with deleted exon 10, and seamlessly joined exon 9 and exon 11 for further research. Meanwhile, He et al. (2021) used paired gRNAs and CRISPR-Cas9-mediated HR to replace the abnormal CAG expansions (74 CAG) with normal repeats (17 CAG) [133]. He et al. reached 1.7% efficiency, while the efficiency of the genome editing performed by Ouyang et al. is unknown. In both studies, the normal karyotype and retained pluripotency of the modified iPSCs were confirmed. Moreover, no changes were detected at the top-ten off-target sites proposed by in silico analysis. Correction of the mutant *ATXN3* allele was verified by RT-PCR and Sanger sequencing [132] or a western blot [132,133]. In addition, He et al. found a lack of significant CNVs or genomic changes with whole-genome sequencing [133]. Yet, Ouyang et al. proved that the CAG tract was stable, while the truncated ataxin-3 protein failed to aggregate and remained susceptible to ubiquitin binding. Corrected SCA3-iPSCs were differentiated into NSCs and neuronal cells in both studies. However, He et al. generated a wider array of mature neuronal cells, including cortical neurons, Purkinje cells and astrocytes. Ouyang et al. showed that in corrected neurons, the mitochondrial function was improved [132]. This was in line with research conducted by He et al., who demonstrated that phenotypic abnormalities such as polyQ protein aggregation decreased the mitochondrial membrane potential, lowered glutathione expression and increased the ROS, while the intracellular Ca2+ concentrations and lipid peroxidase malondialdehyde levels were ameliorated in corrected SCA3 neurons. Moreover, isogenic SCA3 neurons created by He et al. maintained their electrophysiological characteristics [133].

In the context of iPSC editing, besides deleting or correcting the polyQ sequence, insertion of the sequence may also be performed. The same group that pioneered the modeling by HR in polyQ diseases, in 2014, used CRISPR-mediated HR to introduce a 97 mixed CAG/CAA repeat sequence into patient-derived iPSCs with 72 CAG repeats [62]. The use of two gRNA sequences to guide the Cas9 nuclease led to an efficiency level of 12%, which was remarkably higher than the frequency achieved by traditional HR in their previous study (1%) [62,131]. An et al. performed PCR screening to detect the endogenous polyQ-coding region and insert 97 CAG repeats. Clones showing both the loss of an endogenous allele and the gain of an expanded 97Q allele were tested by western blot analysis with a polyQ-specific antibody and Southern blot analysis to confirm targeted recombination [62].

iPSCs are themselves a good model for the phenotypic changes of repeat expansion diseases, though in most cases, they are frequently an intermediate stage during the generation of specialized cells affected by the disease. Differentiation of iPSCs provides human neuronal models that are otherwise difficult to obtain, and it reveals the role of mutant genes in neuronal development [19,153]. However, iPSC-derived neurons do not develop aging-related features, and strategies for accelerating maturation and aging are needed to induce late-onset symptoms [156]. The prospects of genome-edited polyQ models include the differentiation of iPSCs into a 3D network of interacting cells. As of recently, Conforti et al. have developed the first HD organoid system in which they showed that the mutated *HTT* gene affects neuronal differentiation at the early stages of neurodevelopment, thus influencing the later-life phenotypes of patients [158].

### 4.4. Human Embryonic Kidney 293 Cells

Models such as genetically modified HEK 293T cells have the advantage of simple transfection and high-level transgene expression [159]. Yet, though these models are easy to produce and maintain and provide a convenient basis for experiments, HEK-based models are simplified and lack a full genomic background of the promoter strength, or the full-length gene. HEK 293T cells with polyQ transgenes have been successfully used in drug-screening experiments [91] to analyze the molecular pathways and interactors involved in the pathogenesis of polyQ disease [89], as well as to study trinucleotide expansions [130]. An et al. performed a comprehensive analysis of nuclease-based genome-editing methods in HEK 293F cells in 2014 [62]. They compared TALEN-assisted and CRISPR-Cas9-assisted HR as methods of HD modeling. The rate of HR was similar in both cases, but the application of CRISPR-Cas9 technology resulted in a higher number of clones. In the same study, they also confronted the Cas9 nuclease and its more selective mutant—Cas9n. Western blot analysis showed that both nucleases generated 97 CAG expansion at the *HTT* locus in a similar manner.

Subsequently, HD models were created in the HEK cells (HEK 293T [130], HEK 293 Phoenix [147], HEK 293FT [160]) by introducing polyQ-coding repeats into the *HTT* exon 1 locus by CRISPR-Cas9-mediated HR.

Morozova et al. created a panel of isogenic HD models in the HEK 293 cell line [147]. In one of the models, they introduced CAG repeat tracts measuring 100 to 150 repeats. However, the Phoenix cell line was confirmed by FISH to have two full-length copies of chromosome 4 and translocation of an additional small fragment of the chromosome 4 short arm. Thus, some model cells had more than two, different-length *HTT* alleles. Yet, we must highlight that such a model is an extreme case that does not occur in patients. Ultrastructural and morphometric analyses showed that a 100–150 CAG HD model underwent substantial changes in its cell morphology. Cells were characterized by deformation of mitochondrial structures, irregular shapes, a higher density of organelles and accumulation of small autolysosomes.

Another panel of homozygous HEK 293T HD models with different numbers of CAG repeats (41, 53 or 84 CAG) was created by Dabrowska et al. [130]. They adapted an RNP complex composed of the Cas9 protein and gRNA to cause a biallelic mutation at the *HTT* locus. The generated clones were verified by Sanger sequencing and analysis of the huntingtin transcript and protein levels. Importantly, the model showed one of the typical pathological HD features, the production of aberrant *HTT* transcripts [161].

Dabrowska et al. suggest that these HD models are useful for studying CAG repeat expansions and contractions, aberrant splicing, RAN translation, frameshifting and drug screening [130]. As an example, they demonstrated the possibility of allele-selective and non-allele-selective silencing of the *HTT* gene in a set of isogenic HD cells.

The aforementioned studies have proven that in some cases, HEK cells can successfully replace research on cells obtained from patients. HEK 293-based HD models displayed clinically relevant pathological phenotypes, such as abnormal organelle trafficking, structural changes in mitochondria, accumulation of autophagosomes and lysosomes and production of aberrantly-spliced early intron 1 transcripts, which are also present in patient-derived fibroblasts, mouse HD models and biopsy and postmortem samples from HD patients [130,147].

### 4.5. Yeast Cell Models

Genetically modified yeast models are also valuable cellular models in the study of polyQ diseases. The first yeast model of polyQ disease was generated by Krobitsch et al. in *Saccharomyces cerevisiae* [88]. The model successfully presented a polyQ length-dependent inclusion formation and aggregation. The majority of polyQ models, including yeast HD models, express a short fragment of *HTT* exon 1 with an expanded polyQ tract [162]. Meriin et al. generated an HD model with 25 or 103 CAG repeats in the first exon of the *HTT* gene, which displayed polyQ accumulation, and additional toxicity [163]. As the pathological threshold for the polyQ length is not known in yeast, the incorporated polyQ constructs substantially exceed the threshold lengths in patients [164]. Since yeast models successfully reproduce polyQ aggregation, they were used to study the influence of chaperones and protein-folding machinery on this process [162]. The value of yeast as a model organism is underlined by the fact that some of the findings in yeast were confirmed in other model organisms and human patients [87]. Additionally, yeast models are inexpensive and good for large-scale genetic and pharmacological screening [164].

However, the easy access and rapid development of more human-relevant models reduces the importance of yeasts in disease modeling. To our knowledge, no yeast polyQ model has been developed using modern nuclease-based genome editing as of yet.

## 5. Animal Models of PolyQ Disease

The main disadvantage of cellular models is that the influence of the immune and endocrine systems, intercellular communication and the effects of inflammation or signaling molecules are not taken into account. Additionally, behavioral phenotypes, which are of key importance in the context of neurodegenerative diseases, cannot be simulated in cellular models. Therefore, animal models showing more advanced phenotypes and typical behaviors are indispensable for polyQ disease modeling.

### 5.1. Simple Model Organisms

Among the simplest models that favored the study of polyQ diseases in the context of the whole organism were those made in *Caenorhabditis elegans* (nematode), *Drosophila melanogaster* (fruit fly) and *Danio rerio* (zebrafish). These models, with the full gene [95,102] or with a truncated gene, were used with the expanded CAG repeat tract [96,97,98]. They convincingly showed pathogenic features of polyQ diseases including aggregate formation, the toxicity of the mutant proteins, neurotransmission defects and progressive neuronal degeneration [92,96,97,98,102,165,166,167]. Additionally, they are excellent models with which to study the mechanisms underlying polyQ disease symptoms, find potential targets for therapeutic interventions, search for new interactomes and verify findings from other models or patients [95,96,97,101,167]. Yet, though these models are still in use and provide valuable insights into polyQ disease, they are slowly becoming obsolete and their popularity is declining. This is probably the reason why nuclease-based genome editing has not been used to model polyQ diseases in these organisms so far.

### 5.2. Rodents

Rodent models have gained the most popularity as animal models of polyQ disease. Among the first, yet still extensively used, rodent models of HD express only the N-terminal HTTs such as R6/2 with approximately 120 CAG repeats [80] or models with full-length HTTs created using yeast artificial chromosome (YAC) technology and bacterial artificial chromosome (BAC) technology [81,82,83,84]. YAC128 HD mice express mutant HTT with 128 CAG repeats [82], whereas BACHD rodents express mutant HTT with 97 CAG repeats [83]. All of these models display severe and visible behavioral phenotypes of HD.

The mouse models of neurodegenerative diseases have many advantages. First, as mammals, mice are more human-related than simpler organisms such as the fruit fly, zebrafish or nematode. Given their accessibility for engineering, propagation and study from the molecular, phenotypic and behavioral sides, rodent models are the best animal models for large cohort studies [168]. Mice have few requirements for housing, a short life span and rapid breeding cycles. Additionally, genome-editing techniques such as CRISPR-Cas9 have been extensively studied for editing the rodent genome. A vast array of behavioral tests is available to assess mice’s movement, mental status and the neurodegeneration process in rodents. Moreover, there are various online resources providing information about rodents’ biology such as genome sequences, anatomy atlases and databases of gene expression or behavior [169]. It must also be noted that murine models often show a great tolerance to the CAG repeat lengths seen in adult patients. To achieve the desired symptoms, the models are usually designed with a far greater number of CAG repeats than those present naturally (97 repeats for BACHD mouse, 128 repeats for YAC128 mouse and 144 repeats for R6 mouse) [154]. Yet, the models differ in terms of the repeats’ stability. CAG expansions in R6/2 mice show high instability while BAC and YAC-generated models are more stable [79]. Incorporating CAA codons into the CAG repeat tract prevents unwanted expansions and contractions of the polyQ tract [170]. Mouse polyQ models are dominantly transgenic animals generated mostly by viral transduction or microinjection of expression vectors. New nuclease-based genome-editing technologies are mostly used in mice as a possible treatment to correct pathogenic polyQ tracts [69,77]. Yang et al. and Oura et al. proposed a CRISPR-Cas9-mediated repair of CAG repeats within the exon 1 *HTT* gene in mouse HD models [137,148]. Though their main aim was to show the potential of this approach for genetic therapy, they simultaneously generated valuable isogenic models with a corrected *HTT* gene.

Yang et al., in 2017, used CRISPR-Cas9 to suppress the mutant *HTT* gene with 140 CAG repeats in the human *HTT* gene by adenoviral transduction of the HD140Q-KI mouse model striatum [148]. A non-allele-specific approach was used to remove N-terminal *HTT* with the polyQ domain, to alleviate HD symptoms in mouse models. The study was based on recent research that proved that depletion of normal *HTT* in adult mouse brains does not affect neuronal viability, animal survival or growth [171], and the notion that the N-terminal region of *HTT* is not essential for early embryonic development [172]. Models were monitored for possible off-targets by whole-genome sequencing and T7E1 assay. No mutations were found in potential off-target loci, but DNA sequencing revealed frameshift mutations around the targeted *HTT* region. This therapeutic approach proposed by Yang et al. successfully ameliorated early neuropathology and depleted aggregates of mutated *HTT* in the striatum (confirmed by western blotting and immunostaining). Neuronal, autophagy and apoptosis markers remained unchanged. Altogether, the application of this method attenuated body weight reductions and alleviated motor deficits while neuronal viability was not affected. Since the basic parameters of the mice remained unchanged after the introduced change, and its disease symptoms were decreased, it can be used as an isogenic knockout control. Yet, it should be noted that the proposed CRISPR-Cas9 approach only targets human *HTT* exon 1 in HD240Q-KI mice, leaving the rest of the endogenous mice’s *HTT* gene unchanged.

In 2020, Yang et al. studied the influence of the N-terminal mutant HTT on disease development using the CRISPR-Cas9 technique to truncate the *HTT* gene at different sites in HD140Q knock-in mice [149]. By removing the *HTT* exon 1 containing 140 CAG repeats, they showed that exon 1 is not necessary for early development, and in-frame deletion of exon 1 does not disrupt the critical functions of HTT. Additionally, Yang et al. generated mouse models expressing truncated, N-terminal HTT consisting of the first 91 or 571 amino acids. Regardless of the truncation site, N-terminal HTT preferentially aggregated in the striatum in an age-dependent manner and triggered the development of similar defects in tested mice. The authors suggest that selective accumulation of N-terminal HTT in the striatum is associated with the age-dependent expression of a chaperone inhibitory protein—HspBP1.

Yang et al. also performed stereotaxic injection of AAV with gRNA on HD model mice that ubiquitously express Cas9 to truncate the HTT gene, as described above. This experiment further supported the hypothesis that truncation of mutated HTT outside exon 1 does not influence the aberrant protein’s accumulation. Importantly, the mouse model expressing both the mutant *HTT* gene with 140 CAG repeats and ubiquitously expressing Cas9 represents a valuable tool in future research for testing different truncating approaches or studying the DNA repair mechanisms involved in CAG repeat tract expansions and contractions. Oura et al. used CRISPR-Cas9 technology with a modified nuclease variant recognizing NGN PAMs: they applied SpCas9-NG to excise CAG repeats in R6/2 mouse-derived ESCs to breed mouse models with a corrected *HTT* gene [137]. Initial experiments were performed on an ESCs with the HD patient-derived *HTT* exon 1 isolated from the R6/2 mouse. The modified ESCs generated more neural cells than the original ES R6/2 cells and showed no *HTT* aggregates. In the second stage of this experiment, modified ESCs with 35–36 CAG repeats or 2 CAG repeats were used to produce chimeric mice by injection into wild-type (WT) ICR embryos. The produced chimeric mice were mated with WT mice to generate an isogenic model with a corrected CAG tract. The efficiencies of the two-hit and one-hit methods were compared by PCR screening. This showed that a single gRNA is as effective in generating an in-frame CAG deletion as paired gRNAs. The advantage of this method is that it does not require donor DNA that may randomly integrate and destroy endogenous genes. The effectiveness of genome editing ranges from 5 to 13%. Of note, gRNAs designed in this study were not allele-specific, so they could affect wild-type functional alleles. Moreover, direct targeting of the CAG repeats posed a high risk of unpredictable cutting and off-targeting, especially when using the NG-Cas9 enzyme. Off-target analysis conducted on HEK 293T cells showed that NG-Cas9 had a similar off-target frequency and higher efficiency than SpCas9. Inducing DSB within the repeat sequence of the *HTT* gene caused a large deletion, probably due to the unstable nature or repetitive cleavage of CAG repeat tracts. Excessive contraction of CAG repeats could be cancerogenic, which can be prevented by ubiquitin tagging to shorten the Cas9 longevity [137].

CAG repeat contraction reversed the HD phenotype completely. Genome-edited isogenic mice gained weight properly when compared to R6/2 mice, and their motor deficits visible as dyskinesia and their tremors were alleviated. In addition, the cerebral atrophy observed in R6/2 mice was improved in the corrected, isogenic models.

Yang et al., in 2020, generated a panel of isogenic mice to study RAN translation, which enables protein translation in all three reading frames and can cause toxicity [150]. They used CRISPR-Cas9-mediated knock-in in the embryos of HD140Q KI mice to create two models. The first did not express *HTT* with polyQ repeats but allowed for RAN translation, while the other expressed N-terminal HTT with polyQ expansion. Western blot analysis showed that no RAN-translated polyAla or polySer peptides were detected in the brain lysates of analyzed mice. Only in the mouse with N-terminal HTT was polyQ expression observed. Additionally, mice models with RAN translation but not expressing CAG repeats showed a similar performance in behavioral tests to WT mice. RAN translation models do not have a typical HD transcriptomic pattern. This study showed that RAN-translated products do not play a major role in the HD pathogenesis. However, the modeling method for RAN-translation studies proposed by Yang et al. could serve as an example for future research. In line with the set of HD modeling animals proposed here, models can be developed to study RAN translation in other diseases.

The variety of models and studies described above indicates that CRISPR-Cas9 technology is a highly flexible tool. In the context of polyQ diseases, not only can models be made with elongated polyQ tracts or with their contraction but also more advanced models with specific alterations can be made to study complex mechanisms related to the disease pathogenesis.

### 5.3. Large Mammals

Despite mouse models frequently being used, larger mammalian organisms have certain advantages that make them superior in polyQ disease modeling. Firstly, large mammals have greater homology with humans than rodents [168]. Additionally, non-human primate, ovine and porcine HD models display phenotypic features that have not been observed in the smaller animal models, such as dystonia and apoptotic cells in the brain [168]. Larger animals live longer (10–35 years), which allows for long-term analysis of potential therapies in terms of their safety and efficacy [168,173]. They can be more susceptible to the neurotoxic effects of mutant *HTT* expression than rodents [168], which allows for the generation of models with more clinically accurate CAG repeat lengths. However, this increased vulnerability may also lead to early postnatal death. The greatest advantage of large animals over small animal models is that smaller organisms do not fully resemble the human nervous system. Thus, in the context of neurodegenerative disease, the use of larger animal models should be considered in advanced clinical trials as the next step when approaching human application. However, large animal models also have certain disadvantages. The main limitations are late and poorly visible pathological behavior, high costs of animal purchase and maintenance, difficulty in generating large research groups due to long gestations and small litter sizes and finally, ethical concerns [174,175].

To this day, large mammalian models of HD and SCA3 have been generated in monkeys [118,119], marmosets [121], minipigs [117], pigs [116] and sheep [115] thanks to lentiviral infection of embryos or injection of modified cells into oocytes. Both full-length human genes and fragments with polyQ tracts have been cloned into large mammal polyQ models. Yet, until now, only one has been generated with the use of CRISPR-Cas9 technology. In 2018, Yan et al. created an HD pig model by CRISPR-Cas9-mediated replacement of pig *HTT* exon 1 with the human exon 1 containing 150 CAG repeats [140]. Modified pig fibroblasts with confirmed insertion of expanded human *HTT* exon1 in the proper locus were used in SCNT to create the first generation of pig models. Including this generation, Yan et al. created three consecutive generations in which all individuals were positive for the mutant *HTT*. The presence of CAG expansions was confirmed by PCR and DNA sequencing. Genotyping revealed that the CAG repeats were unstable, increasing with each successive generation. Western blotting showed the expression of full-length mutant *HTT* as well as fragmented *HTT* products in the brain tissues of model pigs, but not in the tissues of WT pigs. Immunostaining with anti-*HTT* and polyQ-specific antibodies revealed *HTT* aggregates and mutant *HTT* aggregates in the neurons. Modified pigs showed advanced neurodegeneration, characterized by preferential degeneration of MSNs in the striatum, increased reactive gliosis, the presence of reactive astrocytes, degenerated axons and demyelination. Interestingly, different types of neurodegeneration were found in the cortex and striatum. Additionally, pigs displayed visible external phenotypes such as respiratory difficulties, impaired movement and wrinkled, sagging skin. It was the first time that the HD phenotype manifested as changes in the breathing pattern, as previously, changes in the respiratory system had not been found in other animal models.

The approach taken by Yan et al. allowed for relatively quick construction of large-animal models of HD. The use of CRISPR-Cas9 and SCNT favored the generation of non-chimeric models that could pass the change from generation to generation. Among other things, it has been proven that the mutated *HTT* gene is germline transmittable and that the length of the sequence increases in the next generations. This model can be used not only to analyze the pathogenesis and possible treatment but also to study the disease inheritance and progressive changes caused by aging. Over time, analogous models may arise for other polyQ diseases.

## 6. Conclusions

In recent years, significant progress in modeling genetic diseases has been achieved. An important role has been played by the rapid development of tools for directed genome editing. The examples described above show that CRISPR-Cas9 technology has recently become the most versatile method for generating polyQ models in a variety of organisms and cells. In comparison to the previous genome-editing tools, CRISPR-Cas9 is more effective. Additionally, this method can be relatively easily adapted for use in many experiments. By choosing a proper PAM, gRNA and making changes in Cas9 nuclease cutting domains, different effects can be achieved. Moreover, the editing method developed for a given model can be used as a basis for models in other cells or to create models of other diseases. For now, the risk of off-targets remains the biggest challenge. However, the accuracy of CRISPR-Cas9 technology can be controlled by analyzing off-targets. The possible influences of genome editing on the karyotype, genomic stability and cell viability should be also examined. Methods have already been developed that improve the accuracy of CRISPR-Cas9 technology, including some influencing the durability of the Cas9 nuclease or modifying the system to increase its specificity. With time, the progressive advancement of CRISPR-Cas9 technology is expected to lead to further improvements.

The model’s relevance is also influenced by the choice of modeling organism, as the properties of models related to the physiology of a given organism or cell affect the features of the model and the spectrum of its application. Regardless of the valuable insights provided by existing polyQ models, they do not fully resemble the human brain pathology. Yet, due to our limited modeling possibilities, it is permissible for the models to err from being perfect as it is more important that they resemble the disease characteristics in the context of the analyzed hypotheses. Thus, in most cases, a combinatory approach using different models is applied to fully resemble and examine the mechanisms involved in the question of interest, both on the molecular and phenotypic levels. Most often, initial tests are carried out on cells. For this purpose, models created in the HEK 293 line and iPSCs derived from patients’ cells are mainly used. The ability to create induced immortal cells with clinically relevant features, which can be edited with CRISPR-Cas9 and subsequently differentiated into neuronal cells, greatly facilitates polyQ research. Looking ahead, the use of modern genome-editing tools to create isogenic lines or introduce mutated alleles may impact our understanding of polyQ diseases and make a real contribution to developing future therapies.

## Figures and Tables

**Table 1:** Characteristics of polyQ diseases.

Disease	Gene	Locus	Protein	PolyQ Tract Length			Affected Cells	Main Site of Vulnerability	Refs.
				Normal	Intermediate	Disease			
DRPLA	*ATN1*	12p13.31	Atrophin-1	7–35	35–47	49–88	Striatal, medium spiny, pallidal neurons	Basal ganglia, cerebellum, cerebral cortex, subthalamic nuclei	[20,21]
HD	*HTT*	4p16.3	Huntingtin	10–26	27–35	36–250	GABAergic, medium spiny, striatal neurons	Cerebral cortex, striatum	[22,23,24,25]
SBMA	*AR*	Xq12	Androgen receptor	5–34	35–46	37–70	Motor neurons	Brainstem, spinal cord	[26,27]
SCA1	*ATXN1*	6p22.3	Ataxin-1	6–35	36–38	39–91	Purkinje neurons	Brainstem, cerebellum, cerebral cortex, dentate nucleus	[28]
SCA2	*ATXN2*	12q24.12	Ataxin-2	14–31	27–33	33–500	Purkinje neurons	Brainstem, cerebellum, cerebral cortex	[29,30]
SCA3	*ATXN3*	14q32.12	Ataxin-3	11–44	45–59	60–87	Motor neurons	Basal ganglia, brainstem, cerebellum, spinal cord	[31,32]
SCA6	*CACNA1A*	19p13.13	Ca^2+^ channel subunit α1-A	4–18	19	20–33	Purkinje neurons	Cerebellar cortex	[33,34,35]
SCA7	*ATXN7*	3p14.1	Ataxin-7	4–19	28–35	34–460	Retinal, cerebellar, medulla oblongata neurons	Brainstem, cerebellum, retina, visual cortex	[36,37]
SCA17	*TBP*	6q27	TATA-binding protein	25–41	41	46–55	Purkinje, medium spiny, cortical, dopaminergic neurons	Cerebellum, cortex, substantia nigra, striatum	[38,39]

**Table 2:** Summary of polyQ disease models created using genome-editing tools.

Gene	Model Type	Genome	Number of gRNA	Delivery Method	Number of Repeats				Characteristics	Differentiation/ Reprogramming	Observed Changes	Ref.
		Editing Tool			Before	Modified						
*HTT*	Human embryonic fibroblasts	SpCas9	1	Plasmid/ nucleofection	47 CAG	69 CAG			PCR, WB, off-target analysis, CE, karyotyping, RT-qPCR, pluripotency test, IHC, embryoid bodies’ formation, growth factor withdrawal assay, transmission electron microscopy, scanning electron microscopy, morphometric analysis	iPSCs, MSNs	Impaired neural rosette formation; Increased sensitivity to growth factor and BDNF withdrawal; Changes in cell morphology	[146]
*HTT*	RUES2 hESCs	SpCas9	2	Plasmid/ nucleofection	20/22 CAG	45, 50, 58, 67 and 74 CAG			WB, IHC, off-target analysis, sequencing, karyotyping, pluripotency test, array comparative genomic hybridization	NPCs, postmitotic neurons	Differential *HTT* protein distribution; Decreased self-organization of neural rosettes; Reduced fraction of larger rosettes; Dysregulated mitotic spindle orientation; Chromosomal instability; Multiple nuclei; Disorganized filaments and vacuoles; Multiple centrosomes; Aneuploidy	[138]
						Homozygous knockout (*HTT*−/−)						
						Heterozygous knockout (*HTT*+/−)					Intermediate level of neural rosette organization; Mild multinucleated phenotype	
*HTT*	H9 ESCs	TALEN	2	Plasmid/ nucleofection	~19 CAG	30, 45, 65 or 81 CAG			pluripotency test, G-banding analysis, analysis of CNVs, γH2A.X levels’ analysis, WB, IHC, mitochondrial respiration analysis, RT-qPCR, PCR, cell replication analysis, RNA sequencing, whole-proteome analysis	NPCs, neurons, hepatocytes, skeletal muscle myotubes	Mitochondrial dysfunction; Increased ROS levels; Increased DNA damage; Changes in cell proliferation; Differentially expressed genes; Differentially produced proteins	[63]
*HTT*	iPSCs	-	-	Plasmid/ nucleofection	73 CAG	21 or 20 CAG			PCR, Southern blot, *HTT* exon 1 sequencing, WB, IHC, karyotyping, RT-qPCR, TUNEL assay	NSCs, striatal neurons, glial cells	Decreased sensitivity to growth factor withdrawal; Increased maximum respiration; Differentially expressed genes; Higher levels of TGF-β1 and N-cadherin	[131]
*HTT*	iPSCs	SpCas9	1	Plasmid/ nucleofection	19 CAG	97 CAG			PCR, Southern blot, WB	-	-	[62]
					72 CAG	97 CAG						
*HTT*	Human embryonic fibroblasts	SpCas9	1	Plasmid/ nucleofection	47 CAG	69 CAG			PCR, WB, off-target analysis, CE, karyotyping, RT-qPCR, pluripotency test, IHC, embryoid bodies’ formation, growth factor withdrawal assay, transmission electron microscopy, scanning electron microscopy, morphometric analysis	iPSCs, MSNs	Impaired neural rosette formation; Increased sensitivity to growth factor and BDNF withdrawal; Changes to cell morphology	[146]
*HTT*	RUES2 hESCs	SpCas9	2	Plasmid/ nucleofection	20/22 CAG	45, 50, 58, 67 and 74 CAG			WB, IHC, off-target analysis, sequencing, karyotyping, pluripotency test, array comparative genomic hybridization	NPCs, postmitotic neurons	Differential *HTT* protein distribution; Decreased self-organization of neural rosettes; Reduced fraction of larger rosettes; Dysregulated mitotic spindle orientation; Chromosomal instability; Multiple nuclei; Disorganized filaments and vacuoles; Multiple centrosomes; Aneuploidy	[138]
						Homozygous knockout (*HTT*−/−)						
						Heterozygous knockout (*HTT*+/−)					Intermediate level of neural rosette organization; Mild multinucleated phenotype	
*HTT*	H9 ESCs	TALEN	2	Plasmid/ nucleofection	~19 CAG	30, 45, 65 or 81 CAG			pluripotency test, G-banding analysis, analysis of CNVs, γH2A.X levels’ analysis, WB, IHC, mitochondrial respiration analysis, RT-qPCR, fragment sizing, cell replication analysis, RNA sequencing, whole-proteome analysis	NPCs, neurons, hepatocytes, skeletal muscle myotubes	Mitochondrial dysfunction; Increased ROS levels; Increased DNA damage; Changes in cell proliferation; Differentially expressed genes; Differentially produced proteins	[63]
*HTT*	iPSCs	-	-	Plasmid/ nucleofection	73 CAG	21 or 20 CAG			PCR, Southern blot, *HTT* exon 1 sequencing, WB, IHC, karyotyping, RT-qPCR, TUNEL assay	NSCs, striatal neurons, glial cells	Decreased sensitivity to growth factor withdrawal; Increased maximum respiration; Differentially expressed genes; Higher levels of TGF-β1 and N-cadherin	[131]
*HTT*	iPSCs	SpCas9	1	Plasmid/ nucleofection	19 CAG	97 CAG			PCR, Southern blot, WB	-	-	[62]
					72 CAG	97 CAG						
*HTT*	iPSCs	SpCas9n	2	Plasmid/ nucleofection	180 CAG	18 CAG			PCR, WB, sequencing, off-target analysis, IHC, karyotyping	forebrain neural cells	Improved neural rosette formation; Decreased sensitivity to growth factor withdrawal; Differentially expressed genes; Improved mitochondrial respiration	[139]
*HTT*	iPSCs	SpCas9	2	RNP complex	19 CAG	Knockout			sequencing, pluripotency test, karyotyping, off-target analysis, WB, RT-qPCR	-	-	[130]
			1		19/109 CAG	19/19 CAG						
*HTT*	HEK 293T	SpCas9	1	RNP complex	19 CAG	41, 53 or 84 CAG			PCR, RT-qPCR, WB, sequencing	-	-	[130]
*HTT*	HEK 293 Phoenix cells	SpCas9	2	Plasmid/ nucleofection	~19 CAG	100 and 150 CAG			PCR, RT-qPCR, WB, IHC, real-time cell analysis, viability assay, FISH, electron microscopy, morphometric analysis	NSCs and neuronal cells	Increased apoptosis; Higher organelle density; Changed mitochondrial morphology; Autolysosomes	[147]
						~16 CAG					Changed mitochondrial morphology; Autolysosomes; Autophagosomes; Changed Golgi apparatus and rough ER; Accumulation of lipid droplets	
						Knockout (shifted reading frame)						
*HTT*	Mouse	SpCas9	2	AAV vector/ injection into striatum	140 CAG	CAG repeats removed			WB, IHC, sequencing,	-	Decreased striatal *HTT* aggregates; Reduced astrocyte reactivity; Increased weight gain; Alleviated motor deficits	[148]
									T7E1 assay			
*HTT*	Mouse	SpCas9	1	mRNA/ injection into embryos	140 CAG	Exon 1 removed			WB, behavioral tests (rotarod performance, balance beam test), RT-qPCR, sequencing	-	Development and motor functions similar to WT mice	[149]
						140 CAG (HTT truncated to 96 aa)			PCR, RT-qPCR, WB, (rotarod performance, balance beam test, grip strength test), RNA sequencing, IHC, T7E1 assay		Striatal aggregation of mutant *HTT* in an age-dependent manner, impaired motor functions (in comparison with WT mice)	
						140 CAG (HTT truncated to 571 aa)						
*HTT*	Mouse	SpCas9	1	mRNA/ microinjection	140 CAG	RAN products, no *HTT* with CAG expansion			WB, IHC, behavioral tests (rotarod performance, balance beam test, grip strength test), RT-qPCR, RNA sequencing	-	-	[150]
						Only N-terminal *HTT* with 140 CAG					Impaired movement; Reactive astrocytes; Microglia activation; Differentially expressed genes	
*HTT*	Mouse	SpCas9-NG	1 or 2	Plasmid/ nucleofection; injection into embryos	140–147 CAG	35–36 CAG or 2 CAG			PCR, sequencing, off-target analysis, indel analysis, embryoid body formation, IHC, H&E staining	-	Increased number of neural cells; No huntingtin aggregates; Increased body weight gain; Improved longevity; Decreased tremors; Improved movement; Alleviated cerebral atrophy	[137]
*HTT*	Pig	SpCas9	2	Plasmid/ nucleofection; SCNT	~18 CAG	150 CAG			PCR, sequencing, off-target analysis, WB, IHC, behavioral analysis, ultrastructural analysis (electron microscopy), magnetic resonance imaging, stereology	-	Fragmented *HTT* products; Brain region-dependent mutant *HTT* expression; Decreased weight gain; Wrinkled and sagging skin; Walking abnormalities; Unstable CAG repeats; Dysregulation of the respiratory system; Reactive astrocytes and microglial cells; Reduced brain size; Reduced number of MSNs; Degenerated organelles; Swollen mitochondria; Axonal degeneration	[151]
*ATXN2*	iPSCs	SpCas9	1	Plasmid/ nucleofection	44 CAG	22 CAG			genotyping, sequencing, RT-qPCR, IHC, embryoid body formation, karyotyping	-	-	[134]
*ATXN2*	iPSCs	SpCas9	1	Plasmid/ nucleofection	44 CAG	22 CAG			genotyping, sequencing, RT-qPCR, IHC, embryoid body formation, karyotyping	-	-	[135]
*ATXN2*	iPSCs	SpCas9	1	Plasmid/ nucleofection	36 CAG	22 CAG			genotyping, sequencing, RT-qPCR, IHC, embryoid body formation, karyotyping	-	-	[136]
*ATXN3*	iPSCs	SpCas9	2	Plasmid/ nucleofection	26/78 CAG	CAG			PCR, sequencing, RT-qPCR, WB, IHC, ubiquitin binding assay, embryoid body formation, genotyping, pluripotency test, T7E1 assay, chromosomal microarray, karyotyping, mitochondrial respiration tests	NSCs, neural cells	Improved mitochondrial respiration	[132]
*ATXN3*	iPSCs	SpCas9	2	Plasmid/ nucleofection	74 CAG	17 CAG			off-target analysis, sequencing, T7E1 assay, PCR, WB, CE, IHC, FC, RT-qPCR, in vivo teratoma test, mitochondrial membrane potential test, electrophysiological tests	NSCs, cerebral cortical neurons, Purkinje progenitor cells	Maintained electrophysiological characteristics; No polyQ aggregates; Improved mitochondrial respiration; Decreased mitochondrial membrane potential; Decreased glutathione expression; Increased ROS levels; Increased Ca2+ concentrations; Increased MDA levels	[133]

Aa—amino acids, AAV—adeno-associated virus; BDNF—brain-derived neurotrophic factor; CE—capillary electrophoresis; ER—endoplasmic reticulum; FC—flow cytometry; FISH—fluorescence in situ hybridization; H&E—hematoxylin and eosin; IHC—immunohistochemistry; MDA—malondialdehyde; NSCs—neural stem cells; RAN – **r**epeat-**a**ssociated **n**on-AUG translation; RT-qPCR—reverse transcription-quantitative polymerase chain reaction; SCNT—somatic cell nuclear transfer; T7E1—T7 endonuclease I; TGF-β1—transforming growth factor β1; WB—western blot, WT—wild type.
